# The journey of sensemaking and identity construction in the aftermath of trauma: Peer support as a vehicle for coconstruction

**DOI:** 10.1002/jcop.22373

**Published:** 2020-05-10

**Authors:** Pien van de Ven

**Affiliations:** ^1^ Victim Support the Netherlands & Netherlands Institute for the Study of Crime and Law Enforcement (NSCR: Nederlands Studiecentrum voor Criminaliteit en Rechtshandhaving) De Boelelaan 1077 Amsterdam 1081 HV The Netherlands

**Keywords:** community, humans, interpersonal relations, observation, peer group, trauma, victimization

## Abstract

Sensemaking is rooted in identity construction and it is a particularly interpersonal process. Moreover, traumatic experiences are known to cause people to engage in sensemaking processes and identity construction. However, knowledge of how this works in an interpersonal, community setting, is lacking. The aim of this study is to assess how peer support contributes to the sensemaking processes and identity construction in the aftermath of trauma. Data from an observational study of organised peer support groups for (co)victims of serious crimes and survivors of traumatic loss were analysed using inductive thematic analysis. Results show how participants of peer support groups move through several phases of sensemaking and identity construction in a fluid, dynamic, way. Identity work is collectively done. Through coconstruction of their identities, participants are able to make sense of a traumatic experience and progress towards a more self‐aware and self‐centred identity.

## 1 INTRODUCTION

It is generally acknowledged that sensemaking is rooted in identity construction (Weick, 1995) and that individual's sensemaking of life is influenced by “individual‐specific needs for self‐enhancement, self‐esteem, self‐efficacy, and self‐consistency” (Brown, Stacey, & Nandhakumar, 2008, p. 1040). Sensemaking refers to “processes of interpretation and meaning production whereby individuals and groups interpret and reflect on phenomena” (Brown et al., 2008, p. 1038). Partaking in these sensemaking processes and identity construction is a particularly interpersonal process as it is a cocreation between individuals and communities (Lindemann‐Nelson, 2001; Mankowski & Rappaport, 2000). Moreover, storytelling is the primary interpersonal way people construct personal identities (Gubrium & Holstein, 2000). Especially in the case of negative life events, which require social sharing and narrative attention, an explanation, in one's life story (Pemberton, Mulder, & Aarten, 2019b; Rimé, 2009). While this is widely acknowledged, processes of sensemaking and identity construction after enduring trauma is currently under‐researched and lacks empirical evidence. Particularly considering community settings and how group processes work in sensemaking and constructing identity. As recognisable stories can be powerful tools for identity construction (Copes & Ragland, 2016), such community settings with relatable others lend themselves to identity construction. This study will, therefore, focus on a particular community setting in which stories are told and constructed, namely peer support groups for (co)victims of crime and survivors of traumatic loss. 

A traumatic experience, such as serious victimisation and traumatic loss, can prompt existential questions as fundamental assumptions might be shattered (Janoff‐Bulman, 1992). This causes the individual to feel like they have lost a sense of order in and control over one's life story and the continuation of it, which causes victims and survivors to experience life not as self‐evidently like before (Pemberton, Aarten, & Mulder, 2019a). They ask questions such as “Why me?”, “What has happened to me?”, “What was my role in what happened?” and “How will I be able to move on?”. Making sense of the traumatic experience is necessary to move beyond this experience and move on in life (Park, 2010). Such a traumatic experience thus is a disruption to the victim's or survivor's life story, that is their notions of identity, which poses a threat to their sense of self as before the experience the life story created unity, meaning, and goals in life (Crossley, 2000). According to Zehr (2001, p. 189–190): “(v)ictimization represents a profound crisis of identity and meaning, an attack on oneself as an autonomous but related individual in an orderly world so we must recover a redeeming narrative which reconstructs a sense of meaning and identity.”

Moreover, the disconnection between past and present sense of self is likely to impair the connection and coherence between the individual's narrative and that of the social surroundings (Pemberton et al., 2019b). This disconnection is likely to cause the individual to experience uncertainty, doubt and shame regarding his or her perspective (Brison, 2002). As Brison (2002, p. 51) describes: “In order to construct self‐narratives we need not only the words with which to tell our stories, but also an audience able and willing to hear us and to understand our words as we intend them. This aspect of remaking a self in the aftermath of trauma highlights the dependency of the self on others and helps to explain why it is so difficult for survivors to recover when others are unwilling to listen to what they endured.” Then, together with sensemaking as previously discussed, reconnection is of utmost importance for people who have endured a traumatic experience. Narratives built through sharing, construct, and sustain social connection through reconstructing identity and learning how to cope with a sense of togetherness (Glazer & Marcum, 2003). Therefore, peer support can be of contribution to the success of victims' and survivors' attempts to cope (e.g., Maercker & Horn, 2013; Rappaport, 1993).

The key question the current article seeks to answer is how peer support contributes to the sensemaking processes and identity construction in the aftermath of a traumatic experience. By finding answers to this question we aim to contribute to a new perspective on the role of peer support in the aftermath of trauma in three ways. First, through studying stories and the creation thereof, we take on a narrative perspective. By doing so, this study moves away from the current positivist approach to peer support (Dadich, 2009; Van de Ven, 2018) and looks at it as a vehicle for identity change and reconstruction. This is in line with the current stream of and demand for empirical research in narrative victimology and criminology (Hourigan, 2019; Pemberton et al., 2019b; Presser, 2009; Presser & Sandberg, 2015; Sandberg, 2010; Sandberg & Ugelvik, 2016; Walklate, Maher, McColloch, Fitz‐Gibbon, & Beavis, 2019). Second, through this study, we follow up on Pemberton et al.'s (2019b) request to broaden the research focus in victimology from merely the traumatic experience to the way such an experience can be embedded in the construction and perception of one's identity (see also Rock, 2002). By this expansion of focus, they suggest that much may be learned about the influence of a traumatic experience on perceptions of time, morality, and the self (Pemberton et al, 2019b; see also Cook & Walklate, 2019). We add to that also by looking into the role of community in such recovery processes. And lastly, we bring rather new, and uniquely valuable, empirical perspectives to the stream of narrative research by using data from an observational study, rather than commonly used interviews (Fleetwood & Sandberg, 2019; Fleetwood, Presser, Sandberg, & Ugelvik, 2019; Hourigan, 2019).

To answer the main question, this study presents the results of a unique observational study of organised peer support groups. To explore the issues at hand, this paper falls into four parts. First, a further discussion of what peer support is and how it is currently researched will follow. Second, the methods of the observational study and analysis will be outlined. Third, findings from this observational data will be presented and last these findings will be discussed together with limitations and implications of the study.

### 1.1 Peer support as vehicle for identity construction

In general, peer support is about sharing emotions and experiences through story exchange with people who have endured a similar experience (MacNeil & Mead, 2005; Solomon, 2004). It is based on principles such as respect, trust, shared responsibilities and mutual support (Burke et al., 2018; Mead & MacNeil, 2006; Salzer, McFadden, & Rappaport, 1994; Solomon, 2004). Peer support is not based on models of treatment or therapy but rather takes the similar experience as a starting point for creating empathic understanding and mutual support (see also Mead & MacNeil, 2006; Mead, Hilton, & Curtis, 2001; Rappaport, 1993). Peer support is available in many forms such as support groups, internet fora, drop‐in houses, creative activities, outings, lectures by experts, and commemoration days. It can be led by professionals or peers, or both, and they could be trained or untrained for this specific type of support. It can fit anywhere on the continuum between structured programs only open to a particular set of participants and never‐ending settings open to anyone with a similar experience who is interested. Peer support, therefore, is an elastic, catch‐all concept (see also Van de Ven, 2018). Through narrating their experiences, peers share their experiential knowledge: unique and pragmatic specialised information and perspectives stemming from the lived experience (MacNeil & Mead, 2005; Borkman in Lagrand, 1991, p. 212; Seebohm et al., 2013). They, therefore, together create an arena where processes of social comparison normalise the individual's perspective, provide possibilities for reframing the experience, serve as a platform to learn new coping strategies and fulfill the need for self‐development (Festinger, 1954; Rappaport, 1993; Schachter, 1959; Schutt & Rogers, 2009; Taylor et al., 2007). Processes of mutual aid then enhance feelings of self‐worth and general feelings of wellbeing (Riessman, 1965; Skovholt, 1974). Therefore, contact with people who have a similar experience, and thus are familiar with the accompanying questions and emotions, can be helpful in the aftermath of trauma. The development and delivery of narratives about the traumatic experience can serve as a crucial aspect of the recovery process in the aftermath of such an experience (Hourigan, 2019). Participants in a peer support setting then share the traumatic experience as a “referent”, “a common experience, about which they may infer different meanings but which continues to tie those understandings together” (Weick, 1995, p. 75).

Research into peer support mainly comes from the field of medicine and mental health care and usually take a strong positivist outlook on peer support (Dadich, 2009; Humpreys & Rappaport, 1994; Van de Ven, 2018). This is a rather abstract, instrumental, and quantitative approach measuring the “effectiveness” in terms of outcome variables such as better health and wellbeing. However, by strictly using outcome variables to measure the effectiveness, the current approach to researching peer does not do justice to the collective experience of narrating and connecting. As peer support is a narrative practice designed so that people collectively look for answers to existential questions, such a positivist outlook teaches us little about the way in which processes of sensemaking and identity construction unfold and the way in which time plays a role in this. As a result, empirical studies into peer support for people who have endured a traumatic experience of injustice are lacking (Van de Ven, 2018). As Pemberton et al (2019b, p. 17) put it: “research on peer support still seems to suffer from the presumption that its ‘effectiveness’ must be defined and measured using strict demarcations. Hence, very little is currently known about the processes and interactions within peer support for victims of crime.” Thus, rather than primarily gaining better health and wellbeing, peer support is about making sense of the traumatic experience and incorporating this experience in a newly, coconstructed, identity and restore a sense of normalcy and predictability in life (Hourigan, 2019). As Cain, who thoroughly studied AA meetings, which are considered as a form of peer support, (1991, p. 215) puts it: “[…] it is a cognitive tool, a mediating device for self‐understanding and the stories told are a vehicle for identity acquisition.” She, however, also acknowledges that ethical issues involved in transforming a person's identity through community values should be considered as one's perception of self is taken away and is replaced with an identity that is different (Cain, 1991).

## 2 METHODS

### 2.1 Setting

The data analysed in this article are derived from an observational study of peer support groups in an organised and formalised setting. As peer support groups are a narrative practice, an observational design fits this study perfectly, since, as Hourigan (2019, p. 261) puts it: “studies observing storytelling in situ are able to explore what stories do in those social settings and for the people that tell them.” It enables the researcher to witness the construction and use of narrative in a community setting. Seven support groups were observed during their voluntary participation in a peer support group provided by a victim support organisation in the Netherlands. The observed groups are three groups of child sexual abuse victims, one group of parents of sexually abused children, one group of bereaved parents who lost their child through suicide, one group of bereaved partners who lost their partner through suicide, and one group of bereaved parents who lost their child through a traffic accident. In total, six meetings of about 2.5 hr, which took place about every other week, with a possibility for one extra meeting if necessary. Groups consisted of four to nine participants who were all screened by the group facilitators before taking part in the group. Screenings were done by two group facilitators to assess the individual's capability to participate based on social skills, their ability to both share and listen, and dependency on drugs or suffering from psychopathological characteristics. For the matter of identity acquisition, it is important to note that no preexisting program, like a 12‐step program in AA settings, was used. Themes to be discussed during the group meetings were mainly determined by participants themselves, dependent on requests and needs of the group, and guided by the facilitators. Therefore, all input for telling and creating stories, that is sensemaking and identity work, came from participants' own initiative. All group facilitators were employees and volunteers of the victim support organisation and were specifically trained for guiding these kinds of support groups.

Inevitably, there are differences between the groups because of the type of traumatic experience, group composition, and the facilitators' style of guiding the group. Nevertheless, as these differences do not answer to our main questions, we will not discuss these issues in this paper. We will, however, indicate major differences by providing illustrative examples as appropriate.

### 2.2 Data collection and analysis

Access for the researcher to observe the groups, and thus the type of traumatic experience that brought the group together, was dependent on internal infrastructure of the victim support organisation, willingness for cooperation of group facilitators and permission of all participants. Participants gave oral consent for the observations during the screening with group facilitators who registered their consent. During the first meeting, the researcher introduced herself and the research and gained participants' oral consent again. To give participants choice and control they were explicitly given the option, both in writing before the meetings and orally during the meetings, of asking the researcher, either directly or via the group facilitators, to leave the group and to end the research into this particular group. This study gained ethical clearance from Tilburg Law School (TLS‐ERB#2018/06).

Observations were done over the course of 14 months from December 2017 till February 2019 and resulted in 41 reports. Reports are a transcript of what is said by whom, the interactions taking place and the emotions, such as anger, sadness, and joy, which played a role. Quotes presented in this paper are taken from these reports. These quotes are as “accurate as my ear, memory and notes allow” (van Maanen, 2015, p. 158). In this, and in translations of the quotes, the researcher stayed as close as possible to the words used by participants. Long and detailed accounts of personal events were shortly mentioned but not quoted in the reports. To monitor the researcher's impressions of the groups, each report ends with a reflection, written by the researcher directly after observing a meeting. In this, the researcher answers reflective questions about, for example, the general atmosphere of the meeting, what stood out, how she was feeling, how participants reacted to her presence and what distractions she came across.

During the observations, the researcher witnessed the development of individual participants and of the group as a whole. Moreover, the reflective parts of the reports indicated such changes as well. This initiated the idea of creating a developmental framework (see e.g., also Cain, 1991), which is presented in the findings below and can be found in further detail in the supporting information. Using MaxQDA 2018 software, this framework was developed while going through the reports in chronological order, identifying developmental characteristics. This is in line with an inductive thematic analysis as explained by Braun and Clarke (2006; 2012). Following their steps of a thematic analysis, we built from first‐order concepts to the phases explained below. This analysis is according to the thematic narrative analysis as explained by Riessman (2010) as the emphasis of the analysis is rather on the content of what is said and not on the how it is said. The focus of this study is therefore on the (co)construction of stories about themes relating to the aftermath of trauma, and its accessory existential crises, rather than looking into the way stories are narrated.

Out of 51 participants that started the program, 46 finished it. The decision to no longer participate in the program was always on participant's own initiative and reasons for stopping were usually because they felt participating was too soon in the recovery process, too intense or they preferred therapy over the support group. The major part of the participants were female, namely 39 (76.5%). The age of participants lies between 18 and 78 with an average age of 45.

## 3 FINDINGS

Findings are presented under the key phases of sensemaking and identity construction derived from the analysis. To provide insight into sensemaking and identity construction processes in peer support groups, exemplary interactions between participants are stated in the following. Moreover, quotes by individuals reflecting on the interactions in the group are used. The supporting information Table S1 provides an extensive insight into the gathered data and how the interactions and quotes were divided over the phases after analysis.

From the analysis, a journey appeared in which victims and survivors travel through several places (phases), have encounters (group meetings) with fellow travelers (people with a similar traumatic experience) and share about their individual journeys (of recovery). In this, they provide each other tips and help (experiential knowledge) for the next places to visit. They visit the most beautiful places they have never seen before (new and useful insights and coping strategies), but also come across some stumble blocks (unresolved questions and negative emotions). Through sharing and listening, travelers (participants) find their way (making sense) and coconstruct their stories (identities).

### 3.1 “The world is not ready for our stories”

The first part of the journey is centred around getting to know each other and becoming acquainted with others with similar experiences. Although some participants experience initial resistance to come, and to keep coming, they simultaneously find out that they are not alone. For participants this can be a breakthrough in the isolation felt before meeting these people and motivates them to return to the meetings. Through listening to similar stories and asking questions, peers create normalisation and experience recognition. Also, through sharing their experiential knowledge, their peers provide them with a new frame of reference, valuable and concrete advice, and narratives that teach them how to cope, which were not available to them anywhere else. Bonds, connections, and trust are established in this first phase, which are critical for the course of the rest of the journey. The following interaction shows how participants validate each other's feelings and actions, sometimes unknowingly, by telling about their own experiences. Letters A to D are used to indicate speakers in an interaction. GF is used to indicate when a group facilitator is speaking:

“A: You remain very vulnerable.

B: Sometimes you feel good, then you just don't want to talk about it.

GF: You don't want to talk about it, but does that mean that you don't do it?

B: We sometimes do our grocery shopping in a different supermarket to avoid people.

C: We once hid behind the vegetables for a long time [laughs and tells the story].

D: Ahh, it's one of those people; very nosy and impossible to evade.

A: But this is great because I thought it was weird to go to a different supermarket. But apparently you do it as well.” (Suicide—parents, second meeting)

Participant B answers the group facilitator's question whether participant A act upon her needs through providing an example how she acts upon the same need, namely to sometimes just not wanting to talk about the loss of their child. This not only normalises participant A's decision to do her grocery shopping in a different supermarket than usual, which she thought was weird. It also validates experiencing the feeling of not always wanting to talk about the loss or sharing it with everyone.

Also, in this phase, a feeling of unity is created through othering the outside world. Peers start to sense they are accepted and becoming part of a fellowship. They sense their equality despite the variations in their stories, feelings and progress in recovery. When asked about their experiences of the first meeting, one of the participants stated:

“I have very mixed feelings about it. A lot is dredged up here and I now see what I need to do. But you can really say anything without being judged. I've been judged so many times. Before I came here, I decided I wouldn't talk about anything, just listen. However, when I started talking, I realised that here it is for the first time that I wasn't judged for the fact that my daughter is under legal supervision.” (Parent sexually abused child, second meeting)

This statement was followed by other participants' affirmative nodding and supportive comments. Such a statement shows how this participant started trusting the group, and felt safe to talk instead of just listening, as they did not judge her. Such judgement had occurred outside of the group, where others viewed the legal supervision of her daughter as a failure of her qualities as a mother. The affirmations then show that other participants understand and relate to the statement. Thus, the similar experience establishes a bond as peers understand the challenges of the aftermath of trauma, which, according to participants, cannot be understood by people that have not experienced it. This lack of understanding from others on the one hand and the development of a relation of trust and sense of connection and equality on the other hand establishes a normalisation of the trauma narrative amongst the peers. This leads to collectively working on reframing experiences.

Moreover, normalisation also takes place through the realisation of not being the only one experiencing complex thoughts and emotions, such as shame and self‐blame, in the aftermath of trauma:

“A: It's very heavy, every time you talk about it. With the other stories I think it's a good fit.

B: Very heavy, your stories, your photos, it's so sad, it really hurts me. It's really nice, you feel the sadness but it's also nice. That might sound weird, but because you feel the same, it's very nice.

C: It's nice to see these emotions in others, then you're not alone in it and you consent more.

D: I really dreaded coming here. You can talk about it with family and friends, but you can't talk about it for hours. Here, I feel your world has stopped as well. That recognition is nice. Of course I prefer walking outside and having someone else in here, but it is very nice to not be alone in this.

E: Talking like this is very difficult. Everybody does this differently. It does feel good. It is a sort of safety net. And you're still considering it [death of child and aftermath], that is allowed here. That is the main goal of these conversations.” (Suicide—parents, first meeting)

### 3.2 “Do you think I've come to the right place here?”

While getting acquainted with each other and building a relationship of trust, peers are still looking for their place in this collective. A second phase is characterised by individuals exploring the mutual trust and whether this type of sharing and support is in line with their needs. Some participants might decide to look for other ways of making sense of the traumatic experience. During this study, in total six participants decided to stop participating. One of the participants asked the group facilitators:

“Do you think I've come to the right place here? The other stories are so heavy. And mainly a heavy burden to themselves. I don't have that, I don't experience it like that.” (Child sexual abuse, second meeting)

In this, the participant shows how he is downgrading his traumatic experience in relation to others' experiences. In this way, he is exploring both the validity of his own experience and of his value to the group. In this phase of sensing and exploring, sometimes, a comparison of suffering takes place through which participants try to establish the value of their experiential knowledge to the group. Such a comparison, and reactions thereto, might enlighten participants on the developments they go through by hearing from others. They seem to struggle with their held beliefs that are now contested by others. For example, one of the participants repeatedly emphasised how she felt like she had lost a purpose in life after the loss of her only child. She said to another participant:

“A: You still have another child. At least you still have someone.

B: Yeah, but you also feel the pain of the other child.” (Traffic accident—parents, first meeting)

Through such a statement, Participant A shows how she compares her situation to that of the Participant B and, therefore, places herself in a different, entitled, position, as Participant B still has a child left to live for. Participant B then contests this entitlement through her reaction of bearing the pain of the other child as well as her own pain. Through such comparative interactions, participants test their newly established bonds. By expressing frictions experienced in others' stories and checking fellow participants' reactions, awareness is further fostered.

### 3.3 “People expect so much from you.”

Further along in the process, occurring mostly in the first three meetings, is a phase of finding recognition in each other's' struggles with the interactions with others outside the group. Here, participants stress how they are told, by people that do not share a similar experience, to move on in life and to overcome the traumatic experience. Participants talk about the burden of social and institutional expectations they feel they should live up to outside of the peer support group:

“A: Outside of our family, life goes on in a quick tempo. For us, the loss is immense and outside it fades quickly.

B: The difference in speed is large, but the sharp edges fade. In the beginning I only saw the despair of the death [of child], but now I also see the beautiful memories. That's a nice development.” (Suicide—parents, third meeting)

Outside, they feel pressured as, on the one hand, participants do not want to bother others with their stories and emotions. But on the other hand they lack a feeling of recognition and connection, and therefore will look for gratification from others. Talking about these expectations with their peers might change their views on it. Participants start to recognise these expectations as socially constructed by broader societal processes and, therefore, see it is not necessary for them to live up to the expectations of others. This is a first step towards restoring the initially experienced lack of recognition and connection, outside the group, as peers do recognise and validate each other and their stories. This then, again, furthers the connection between peers.

### 3.4 “Don't you understand there is a hole in my identity?”!

During the second half of the meetings of this ongoing journey of identity construction, the participants move away from the focus on people outside of the group and start to have more self‐focussed conversations. In this, they explore how to reconstruct the self. Their perspectives of themselves and their relationships to others start to change. Where they initially focussed on being different because of their traumatic experience, participants now start to view others as different from them:

“A: It makes me mad. I just think: don't you understand there is a hole in my identity?! As a mother. As a widow. Then I just sometimes wish something very bad for them. It is not nice and not fair, but I would really appreciate their understanding.

B: Yeah, they didn't experience it. That's why I wanted to be here [at peer support group]. I had doubts, but I think you are the only ones that understand. I have people to talk to, but they cannot put themselves in my place. That's why I want to be here, every time. I like it. Sometimes, people say: “it's been a year and a half now, isn't it just done?” It's not necessary to constantly talk about it, but sometimes I want to talk about it.” (Suicide—partners, fifth meeting)

“It is a big emptiness. I cannot relate my experience to others. They expect me to be happy to be pregnant. I am not. I'm just messing about.” (Suicide—partners, fifth meeting)

To participants, others, who have not endured a traumatic experience, are ignorant as they will never understand what people go through after such a life event. Others will, therefore, never understand the disconnection in identity participants experience.

### 3.5 “I could get rid of a very heavy weight off my shoulders”

Besides the acknowledgement of a deficit in one's identity and understanding others will not comprehend this, participants also express feeling relieved through participating in the peer support group. This relief seems to provide a certain clarity through which participants experience a new window of opportunity. This new outlook on certain emotions, thoughts and experiences seems to release participants from the complexity of it enabling them to experience new, lighter and more positive, emotions:

“I am so glad I came here and I am so happy I met you. My family tells me I have changed. I feel better in my head. I am not there yet, but before I came here, I had no idea where to start. I have calmed down and I don't send offensive texts anymore. And I don't cry anymore when I talk about it.” (Parent of sexually abused child, fifth meeting)

Moreover, in most phases of the peer support journey, participants communicate how they have lost a part of themselves through the traumatic experience. The expressed relief in this phase then is also established through finding something of themselves in others:

“The last time we met, I almost couldn't speak. It was so heavy. For us, it's been 16 months now [death of child] and still we feel like every time we walk into this fog. This fog, it's… You lose something of yourself and when you've been here [at the support group] you retrieve it.” (Suicide—parents, second meeting)

### 3.6 “I'm here too.”

The shift towards a more self‐aware outlook, the relief, and the clarity that comes with it, leaves room for participants to focus on both their own wellbeing and the wellbeing of their fellow participants. Conversations at this stage move away from externalising to others and are more focussed on developments participants themselves go through and the challenges they face in these developments, for example in relationships, health or at work. In the following, Partners A and B reflect on how to move on in life after the suicide of their daughter:

“A: This powerless feeling of what else could I have done?

Group facilitator: is that changing?

A: slowly, I'm starting to be a bit more at ease with this.

B: And still it is hard. We were so trapped in the patterns of our daughter that it feels weird to now suddenly do things differently.” (Suicide—parents, fourth meeting)

Other examples are a participant who decided to pause her study and focus on her recovery due to what she learned from a group meeting focussed on self‐care (child sexual abuse, third meeting), a participant who decided to visit an open, 1 day, peer support setting in addition to the organised peer support group (child sexual abuse, seventh meeting), and participants trying new types of therapy or finding a new therapist because of what they learned from others' experiences.

In this new window of opportunity, participants understand the choices and opportunities they have. Such realisations do not necessarily come as a result of participating in a peer support setting only. A peer support setting does, however, provide a venue to share such experiences and where this identity shift is thus expressed and celebrated.

“I used to only take the high speed train. Now I sometimes dare to take a slow train and get off sometimes to experience how good that feels.” [Metaphorical speech] (Child sexual abuse, fourth meeting)

Moreover, by more explicitly supporting each other in their developments, questions and insecurities addressed in the group, participants contribute to each other's stories of recovery. More community focussed conversations start to come into existence.

Participants feel safe and secure enough to experience the social niche they found in which they find time and space to talk with full consideration about the experience and at the same time be of help to others. Through this, they move away from previously held beliefs about their identity and move towards more self‐esteem, self‐efficacy and self‐consistency. At this stage, sharing with more emotion is appropriate as well as monitoring each other's feelings and developments. This is how one of the participants expressed how others provided her with new insights that helped her in her own recovery:

“I'm on my way to feeling better, I'm moving in a direction of acceptance. The first meeting [name peer] told us he had forgiven his father. I couldn't understand that. But I realised it just causes a stagnation of my growth. So I also want that. I have gone through all emotions here, but I did recognise progress in myself because of the comments of others in the group that helped me or made me think.” (Child sexual abuse, seventh meeting)

In this quote, both the dynamic process of identity construction and the contribution of a fellow‐participant becomes clear. It shows how in the beginning this participant did not understand another participant's forgiving. However, as time progressed, she started to understand and incorporated forgiveness into her own story of recovery. Moreover, she states how comments of others formed, thus coconstructed, her new identity, which centers on “feeling better” and “acceptance.”

### 3.7 “You're just searching.”

The last phase of this journey of sensemaking considers transferring the coconstructed identity, the newly gained insights, to the world outside the peer support setting. That is, the reality where participants previously experienced a lack of recognition and connection that led them to participate in a peer support setting in the first place. In this last phase, participants are in between identities and explore how to maintain their newly constructed identity outside of the peer support setting. In this, participants face new challenges as they have to let go of the group, the group facilitators and the safe space they created amongst each other. Also, through the process of peer support, bonds and friendships are created based on this shifted identity, which participants are afraid to lose:

“A: It feels weird to say goodbye.

B: Yeah, that's right. Something is off.

C: It's a small loss again.

A: Yeah, I'm a bit scared we will be alone again after this” (Suicide—parents, sixth meeting)

Moreover, in this last phase, participants work towards transforming the traumatic experience into a life experience. Partaking in a peer support setting and collectively working through the experience is a turning point for participants:

“I learned here, that what I suffer from, what I feel, that it is legitimate and ok and that I am allowed to take that into account.” (Child sexual abuse, eight meeting)

What all participants seemed to experience in the last phase of coconstructing identity, towards the end of the series of meetings, was what Schon (2010) names as being in between identities. Participants then have to grow out of the group identity and work towards incorporating the newly attained identity into their life outside the peer support group. Rather than overcoming the traumatic experience, participants work towards managing the experience and incorporating the newly, coconstructed, identity in the reality outside of the group.

What comes across strongly from the results is the journey of the individual in the group and the role of the group in facilitating coming to terms with what has happened and enabling the individual to move on. In this, the metaphor of a journey, as explained earlier, is helpful to understand such a journey.

## 4 CONCLUSIONS AND DISCUSSION

In conclusion to the question of how peer support contributes to the sensemaking processes and identity construction in the aftermath of a traumatic experience, meeting people with a similar traumatic experience appeared to be of major importance to the cocreation of identity in the aftermath of trauma. In this, the role of a peer support group is to serve as a means towards recovery. Recovery then involves sensemaking of the traumatic experience and its aftermath as well as constructing an accommodating identity. This process of identity construction takes place over time, with the identification process occurring in particular phases. During the course of the peer support meetings, processes of confirmation and normalisation contribute to sensemaking of a traumatic experience and to the construction of posttrauma identity. Through listening and talking to people who have endured a similar traumatic experience, people are able to both gain self‐awareness and help others. Through this cocreation of a recovery narrative, an identity shift takes place. Moreover, partaking in a peer support group offers a sense of belonging which participants felt they had lost in the aftermath of trauma. Schon (2010) refers to this as “the power of identification” where the creation of a recovery narrative is a self‐help strategy in the recovery process and a peer support setting serves as an arena for this purpose. The findings of this study are in line with those of the few other studies identifying phases of growth within peer support settings (Cain, 1991; Coatsworth‐Puspoky, Forchuk, & Ward‐Griffin, 2006; Davidson, Chinman, Sells, & Rowe, 2006; Hourigan, 2019; Schon, 2010).

By taking on a narrative approach, we were able to show that this journey is processual and dynamic, consisting of several phases for the participants (see also Pemberton et al, 2019b). Simultaneously, although the phases evolve over time, the journey is characterised by fluidity. The Table S1 in the supporting information shows in further detail how these phases are centred in certain parts of the journey. We should be aware that individual's' responses and attitudes towards the traumatic experience, its aftermath and their sense of self fluctuate over the course of time (see also Heywood et al, 2019; Hourigan, 2019). And although this process is not linear, the dynamic process of identity construction and sensemaking does lead towards growth. Through the coconstruction of identity, participants gradually progress in their recovery. Or, in the words of Cain (1991, p. 242): “[this] indicates that the stories themselves change over time as the understanding of oneself and one's life changes. The stories reflect changes in self‐understanding.” The traumatic experience is therefore not just in the past but becomes interwoven with participant's ongoing lives (see also Pemberton et al., 2019a; 2019b), or as found by Hourigan (2019) a “new normal.” Partaking in a peer support setting then is a turning point in one's journey of identity work (Goodey, 2000). Or as explained by Cook and Walklate (2019), it is an, or is a collection of, epiphany moment. Apart from it being a dynamic process, it is not experienced in the same manner by all participants. Not all participants go through these phases in a similar tempo and participants might move back and forth between phases. The journey presented in this paper is, therefore, mainly a natural process rather than comprising strictly distinct and consecutive phases. This finding points back to our original critique of dominant work on peer support, which does not, and cannot, capture this process as its focus is on results and outcome variables.

Listening and talking to like‐minded peers fulfills two needs people might have in the aftermath of a traumatic experience. First, it resolves some of the complex questions which stem from a difference in perspective between those who have endured a traumatic experience and those who have not, that dominate the aftermath of the traumatic experience. As explained by Brosi and Rolling (2010), articulating the experience, values and expectations, apart from the cultural dominant narrative, provides the participants with a sense of freedom. Second, it can alleviate the quest for recognition and, therefore, restore the damaged connection, between the individual's narrative and that of the social surroundings that brought them to do collective identity work in the first place. It is those collectively articulated and heard stories of trauma that “can bridge divides and encourage identification across groups of people, offering a platform for understanding, reworking and learning about otherwise dissimilar individuals.” (Cook & Walklate, 2019, p. 248). As these needs are being met in a peer support setting, participants move towards a more self‐aware identity. Such a shift, a reworked and restoried understanding of the self, can be seen as post traumatic growth, the positive side of trauma (Kunst 2010; Tedeschi & Calhoun, 2004). Collectively working through the traumatic experience enables participants to manage the experience as a part of them, as a part of their, posttrauma identity.

Moreover, through unique observational data this study contributes to the understanding of recovery processes of people that have endured traumatic experiences that prompt existential questions. This type of data, placed in a narrative framework, enabled us to draw the process of sensemaking and identity coconstruction in the aftermath of trauma, by identifying the phases such a process consists of. This is a rather new and underexposed perspective in relevant fields, such as the study of peer support, identity transformations, and narrative victimology.

### 4.1 Limitations

Whilst an observational study design was considered the most suitable methodology for this study, it does not go without some disadvantages. As the existing infrastructure of organising peer support groups at a victim support organisation was used to gain access to these groups, this study is subjected to selection biases. The first bias stems from this organisational structure, in which not all clients are automatically made aware of the opportunity to participate in a peer support setting. Therefore, those who are not reached by the victim support organisation are, by design, left out of the study. The second bias is a self‐selection bias of participants. Those clients that decide not to take part in peer support groups and those who do not seek for such an opportunity, are automatically not included in this study. In this, it seems that not all people who have endured a traumatic experience feel the need to seek out for help, recognition and validation from others or seek out ways to make sense of the traumatic experience. This study, therefore, only represents one side of the spectrum of responses to peer support, namely those of participants that voluntarily decided to participate. Future research should look into the reasons why people decide to not participate in a peer support setting to further explore this spectrum. Furthermore, it is recommended that future research looks into the differences in processes taking place in peer support settings between the different types of traumatic experiences.

In conclusion, this study underlines the importance of creating a safe space for people who have endured a traumatic experience to be able to share stories, experiences, questions and emotions with people who share in and understand a similar experience and its aftermath. Such a safe space is needed for them to make sense of the experience and find a frame of reference and to reconstruct their identities. In peer support, such identity work is collectively done. Through reframing the experience, participants are able to move forward in their recovery. People who have endured a traumatic experience are made to do identity work, because their lives are fractured by this severe experience. Peer support then might serve as a suitable arena to do such work in a safe and understanding environment.

## CONFLICT OF INTERESTS

The authors declare that there are no conflict of interests.

## ETHICS STATEMENT

This study gained ethical clearance from Tilburg Law School (TLS‐ERB#2018/06).

## Supporting information

Supplementary information

## Data Availability

Data cannot be shared considering the data are too sensitive.
