# Supplementary material for: The journey of sensemaking and identity construction in the aftermath of trauma: Peer support as a vehicle for coconstruction
Source: J Community Psychol. 2020 May 10;48(6):1825–39. doi: 10.1002/jcop.22373 (PMC7496503; doi:10.1002/jcop.22373)
Supplement: Supplementary file 1 — Supplementary information [file JCOP-48-1825-s001.docx]

**Table S1 Interactions and quotes per phase journey through peer support**

| **Characteristics** | **Interactions & quotes** |
| --- | --- |
| **“The world is not ready for our stories.”** | |
| Normalisation, recognition, new frame of reference, othering the outside world, creating bonds.  We all chose to participate, we’re in this together, let’s find a way to trust each other. | “A: You remain very vulnerable  B: Sometimes you feel good, then you just don’t want to talk about it.  GF: You don’t want to talk about it, but does that mean that you don’t do it?  B: We sometimes do our grocery shopping in a different supermarket to avoid people.  C: We once hid behind the vegetables for a long time [laughs and tells the story].  D: Ahh, it’s one of those people; very nosy and impossible to evade.  A: But this is great because I thought it was weird to go to a different supermarket. But apparently you do it as well.” (Suicide parents, second meeting) |
|  | “I have very mixed feelings about it. A lot is dredged up here and I now see what I need to do. But you can really say anything without being judged. I’ve been judged so many times. Before I came here, I decided I wouldn’t talk about anything, just listen. However, when I started talking, I realised that here it is for the first time that I wasn’t judged for the fact that my daughter is under legal supervision.” (Parent sexually abused child, second meeting) |
|  | “A: It’s very heavy, every time you talk about it. With the other stories I think it’s a good fit.  B: Very heavy, your stories, your photos, it’s so sad, it really hurts me. It’s really nice, you feel the sadness but it’s also nice. That might sound weird, but because you feel the same, it’s very nice.  C: It’s nice to see these emotions in others, then you’re not alone in it and you consent more.  D: I really dreaded coming here. You can talk about it with family and friends, but you can’t talk about it for hours. Here, I feel your world has stopped as well. That recognition is nice. Of course I prefer walking outside and having someone else in here, but it is very nice to not be alone in this.  E: Talking like this is very difficult. Everybody does this differently. It does feel good. It is a sort of safety net. And you’re still considering it [death of child and aftermath], that is allowed here. That is the main goal of these conversations.” (Suicide parents, first meeting) |
|  | “People say: you should not be worried to lose your other child. But for them it’s easy to say.” (Suicide - parents, first meeting) |
|  | “The world is not ready for our stories. Here, amongst each other we are ready.” (Historical abuse victim, second meeting) |
|  | “To me, work is really a distraction. What I find really difficult, is going to the canteen. I really do not like the kind of conversations in the canteen. At a canteen table, I feel really awkward.” (Suicide - parents, second meeting) |
|  | A: It’s all just so unpredictable and illogical, all logic is gone.  B: I’m just not myself anymore. You went from ‘parent’ suddenly to a very vulnerable person. How will I ever be able to be happy again? Then I just need someone to tell me it will all be all right. But of course, no one will say that.  A: Your future collapses and you don’t expect it. There would never stand still and realise that.  C: No, it’s also not normal give thought to that, fortunately.  (Suicide - parents, first meeting) |
|  | A: Isn’t that quite hard work, to keep up your appearance every day? B: I’m not used to anything else. I want to live, not survive anymore.  A: Yeah, I recognise that  (CSA, second meeting) |
|  | A: Now, after my therapy, I dare to show emotion.  B: I don’t want to be vulnerable, that is scary. I always had a wall built around me, so that is all new to me.  C: Being vulnerable is dangerous, not even scary.  B: It’s all about being tough, while you actually feel so small.  A: I’m now reading a book about vulnerability. It could also be your strength.  (CSA, first meeting) |
| **“Do you think I’ve come to the right place here?”** | |
| Getting to know each other, experiential knowledge, end of isolation, comparison of suffering, testing the bonds | “Do you think I’ve come to the right place here? The other stories are so heavy. And mainly a heavy burden to themselves. I don’t have that, I don’t experience it like that.” (Historical abuse, second meeting) |
|  | “A: You still have another child. At least you still have someone.  B: Yeah, but you also feel the pain of the other child” (Traffic accident – parents, first meeting) |
|  | “My story doesn’t seem to matter that much when I hear your stories. It makes me think it wasn’t that bad after all.” (Historical abuse, second meeting) |
| **“People expect so much from you.”** | |
| Social expectations, more explicitly supporting each other, change of view on expectations | “A: Outside of our family, life goes on in a quick tempo. For us, the loss is immense and outside it fades quickly.  B: The difference in speed is large, but the sharp edges fade. In the beginning I only saw the despair of the death [of child], but now I also see the beautiful memories. That’s a nice development.” (Suicide - parents, third meeting) |
|  | “People now expect you to be ok. People expect so much from you. I get annoyed by that.” (Suicide partner, second meeting) |
|  | “It makes me really pissed: we did not do anything wrong and still we are seen as abnormal because others find it too difficult to deal with. It is almost as if we are viewed as perpetrators. It is really bad people act like this. They don’t know how to handle it.” (Historical abuse victim, fifth meeting) |
|  | A: Empty. It feels empty. And people saying ‘best wishes’ to each other [on new year’s eve]: it’s just not right.  B: We do have a lot of family. That gives me energy. [she tells a story about going to the grave of her son with the family.] Then time stands still and then time is dedicated to our son. [she and her husband go on telling about celebrating his birthday and about the end-of-year celebrations.] C: There are so many celebration days coming up: new year’s eve, birthday, Christmas, the feast of Saint Nicholas. It makes me panic. I don’t know what to do with all these days. I prefer to lock myself up and appear again when it’s all done.  GF: Can anyone provide tips? Husband of C: we should take it step by step. We shouldn’t look at what is next yet.  [C tells how difficult she finds it all. ‘Everything is different.’] A: You also never really know what you want with these things. C: No really, you don’t. It’s like a rollercoaster.  (Suicide - parents, second meeting) |
| **“Don’t you understand there is a hole in my identity?!”** | |
| More self-focussed, exploring possibilities to reconstruct the self (if possible), others will never understand | “A: It makes me mad. I just think: don’t you understand there is a hole in my identity?! As a mother. As a widow. Then I just sometimes wish something very bad for them. It is not nice and not fair, but I would really appreciate their understanding.  B: Yeah, they didn’t experience it. That’s why I wanted to be here [at peer support group]. I had doubts, but I think you are the only ones that understand. I have people to talk to, but they cannot put themselves in my place. That’s why I want to be here, every time. I like it. Sometimes, people say: ‘it’s been a year and a half now, isn’t it just done?’ It’s not necessary to constantly talk about it, but sometimes I want to talk about it.” (Suicide - partners, fifth meeting) |
|  | “It is a big emptiness. I cannot relate my experience to others. They expect me to be happy to be pregnant. I am not. I’m just messing about (Suicide - partners, fifth meeting) |
| **“I could get rid of a very heavy weight off my shoulders”** | |
| Finding relief and finding something of themselves in others | “I am so glad I came here and I am so happy I met you. My family tells me I have changed. I feel better in my head. I am not there yet, but before I came here, I had no idea where to start. I have calmed down and I don’t send offensive texts anymore. And I don’t cry anymore when I talk about it.” (Parent of sexually abused child, fifth meeting) |
|  | “The last time we met, I almost couldn’t speak. It was so heavy. For us, it’s been 16 months now [death of child] and still we feel like every time we walk into this fog. This fog, it’s… You lose something of yourself and when you’ve been here [at the support group] you retrieve it.” (Suicide - parents, second meeting) |
|  | “Through talking about it and sharing [with the support group] it felt as if I could get rid of a very heavy weight off my shoulders. It was such a relief.” (Historical abuse, fourth meeting) |
|  | A: Aren’t you just mad? I was wondering about that earlier.  B: Yes, I have been mad for three years already. I don’t know how my perpetrators are doing: are they in jail, are they married, etcetera. I’m feeling very sorry about that and I will never find out.  C: It seems difficult to me that you don’t know that. But yeah, will you ever find out? B: I find it very hard that I have all kinds of things [ailments, thoughts, anxiety] which are caused by them.  D: Anger is such an obstruction to your process. You will only keep wondering why.  A: Did you ever express this anger?  D: Yes, once I expressed it very well. And after that, I reported to the police.  A: Yeah, that’s what I mean. You have been able to drop it and feel uplifted.  (CSA, fifth meeting) |
|  | Several peers now talk about sport being helpful for relief  A: underneath that anger is sadness. You should work on that.  B: it’s a matter of finding out what suits you  C: And it’s not always the same things that are helpful  D: And that’s what I find so annoying!  (CSA third meeting) |
| **“I’m here too.”** | |
| More self-aware, focus on own wellbeing and wellbeing of peers, focus on developments, understanding choices and opportunities, shifting identity towards more agentic | ”M: This powerless feeling of what else could I have done?  Group facilitator: is that changing?  M: slowly, I’m starting to be a bit more at ease with this.  F: and still it is hard. We were so trapped in the patterns of our daughter that it feels weird to now suddenly do things differently. (Suicide - parents, fourth meeting) |
|  | “I used to only take the high speed train. Now I sometimes dare to take a slow train and get off sometimes to experience how good that feels.” [metaphorical speech] (Historical abuse, fourth meeting) |
|  | “I’m on my way to feeling better, I’m moving in a direction of acceptance. The first meeting [name peer] told us he had forgiven his father. I couldn’t understand that. But I realised it just causes a stagnation of my growth. So I also want that. I have gone through all emotions here, but I did recognise progress in myself because of the comments of others in the group that helped me or made me think. (Historical abuse, seventh meeting) |
|  | “Someone I was dating now rejects me because of what I endured. It is up to me to decide whether I am ready for a new relationship or not.” (Suicide - partners, sixth meeting) |
|  | “GF: do you directly compare your experiences to those of others, so you learn from them who you are?  A: Yeah, my shame is declining, which makes me more open. But my sister tells me she doesn’t notice any improvements. That’s why I want to keep my distance from her.  B: No, then you shouldn’t use your sister too relate to.  C: We never feel good enough anywhere, that’s what we have in common. That is a pity, that we always think we need others to feel good enough […].  A: My therapist showed me that my sister is weaker, I always looked up to her as my strong and big sister. Then it is difficult to adjust that.  B: Distance yourself from her, she is obstructing your growth.” (Historical abuse, sixth meeting) |
|  | “A: Because of last meeting’s assignment [putting people in a circle of trust] I realised I actually have quite a lot. I’ m too focussed on what I don’t have. That is too bad. I should focus on what I have. Some people do not fit my new ‘me’ anymore. I should keep them at a distance and that is a pity.  B: I think it’s also a recognition for what is happening in your life now.  A: Yeah, I’m really in a process of becoming aware.” (Historical abuse victim, third meeting) |
| **“You’re just searching.”** | |
| Transferring co-constructed identity to outside world. Not about overcoming the experience, but about managing it | “A: It feels weird to say goodbye.  B: Yeah, that’s right. Something is off.  C: It’s a small loss again.  A: Yeah, I’m a bit scared we will be alone again after this” (Suicide parents, sixth meeting) |
|  | “I learned here, that what I suffer from, what I feel, that it is legitimate and ok and that I am allowed to take that into account.” (Historical abuse, eight meeting) |
|  | “Outside of this group, I find it hard to share with others. Here it is easier because I get the feeling that you will provide me with an answer.” (Historical abuse victim, fifth meeting) |
|  | “I notice it makes me think; gosh, soon all of this won’t be here anymore. Even if we keep in touch. You [group facilitators] are here, the building, the setting; it makes me feel safe. That won’t be there anymore after this. I’m really happy with this group.” (Historical abuse, seventh meeting) |
|  | “A chameleon, before I came here, I was a chameleon. I always went where the wind blows Now, I am less concerned with that. Only on moments when I’m having a hard time. I’m going to try to be a different animal. To be able to be more myself and to be able to indicate what I want.” (Historical abuse, eight meeting) |
|  | A: I never thought I would come out of the big black sadness. Right at the moment you take a step out of that you think, ‘Oh, sorry [name of son]’  B: Yeah that’s true, but I think it’s a struggle within ourselves. It’s a boundary we inflict on ourselves. We will never be really happy again. But we can still be happy. We can make it very hard to ourselves by thinking every time that this is not allowed. If we are only sad every day, it will not work out in any way. Deep in your heart you know you don’t relinquish [name of peer’s son]. It is now too early to allow ourselves to be happy.  A: Yeah I think so.  C: You also don’t feel like doing something fun.  B: You have to stay positive and motivated, otherwise you fall down a long way.  A: You want to move on in life for your children. You don’t want them to see you sad.  B: fortunately, it varies. Sometimes one of us [he and his wife] it totally done with it, and at other times it’s the other.  (Suicide - parents, fifth meeting) |
|  | GF: You mentioned Ireland.  A: Yeah, but that is not necessarily for now. We could do that with day trips. I want something for the longer term. I cannot find that yet.  GF: I asked about small steps, just so you can work towards the longer term.  A: That hole should be filled. And that doesn’t work with going to a movie.  GF: Does anyone have any tips?  B: It’s mainly very recognizable. You’ve given so much love and that is now lacking a destination.  A: You’re just searching. One moment you could enjoy buying a small plant.  GF: That is all about your feelings. But what do you do with it? It’s all about letting in those nice moments, regardless of the loss. […] Just think about it. Does that make sense? C: yeah, it does  B: That is really nice, it makes you aware  (Suicide - parents, fifth meeting) |
